# Pheochromocytoma Multisystem Crisis Requiring Temporary Mechanical Circulatory Support: A Narrative Review

**DOI:** 10.3390/jcm14061907

**Published:** 2025-03-12

**Authors:** Raphaël Giraud, Amandine Glauser, Carole Looyens, Chiara Della Badia, Jalal Jolou, Mustafa Cikirikcioglu, Karim Gariani, Karim Bendjelid, Benjamin Assouline

**Affiliations:** 1 Intensive Care Division, Department of Acute Care Medicine, Geneva University Hospitals, 1205 Geneva, Switzerland; raphael.giraud@hug.ch (R.G.); carole.looyens@hug.ch (C.L.); chiara.dellabadia@hug.ch (C.D.B.); karim.bendjelid@hug.ch (K.B.); 2 Department of Anesthesiology, Pharmacology, Intensive care and Emergency Medicine, Faculty of Medicine, University of Geneva, 1205 Geneva, Switzerland; 3 Geneva Hemodynamic Research Group, Faculty of Medicine, University of Geneva, 1205 Geneva, Switzerland; 4 Division of Anesthesiology, Geneva University Hospitals, 1205 Geneva, Switzerland; amandine.glauser@hug.ch; 5 Division of Cardiac Surgery, Department of Surgery, Geneva University Hospitals, 1205 Geneva, Switzerland; jalal.jolou@hug.ch (J.J.); mustafa.cikirikcioglu@hug.ch (M.C.); 6 Division of Endocrinology, Diabetes, Nutrition and Therapeutic Patient Education, Department of Medical Specialties, Geneva University Hospitals, 1205 Geneva, Switzerland; karim.gariani@hug.ch

**Keywords:** t-MCS, VA-ECMO, cardiogenic shock, pheochromocytoma multisystem crisis

## Abstract

**Background:** Pheochromocytoma and paraganglioma are catecholamine-secreting tumors, rarely presenting with pheochromocytoma multisystem crisis (PMC), a life-threatening endocrine emergency. The severity of the condition includes a refractory cardiogenic shock and may therefore require the use of temporary mechanical circulatory support. The aim of this review is to describe the incidence of pheochromocytoma and paraganglioma crises associated with refractory cardiogenic shock, the physiopathological impact of this condition on the myocardial function, the role of temporary mechanical circulatory support (tMCS) in its management, and the outcomes of this specific population. **Methods:** For the purpose of this narrative review, a literature search of PubMed was conducted as of 16 November 2024. Medical Subject Headings (MeSH) terms used included extracorporeal circulation”, “Impella”, “pheochromocytoma”, “paraganglioma”, and “cardiogenic shock”, combined with Boolean “OR” and “AND”. Data from case series, retrospective studies, and systematic reviews were considered. Seven studies reporting on 45 patients who developed PMC with cardiogenic shock requiring tMCS were included. Patients were young, with a median age of 43 years (range 25–65) at presentation. Most cases presented with severe hemodynamic instability, blood pressure lability, and rapid progression to severe left ventricular dysfunction. Veno-arterial extracorporeal membrane oxygenation (VA-ECMO) was the most common tMCS used to stabilize patients, initiate specific pheochromocytoma treatments, and, in some cases, provide circulatory support during emergent surgery. The median duration of VA-ECMO support was 4 days (range 1–7) and the reported mean in-hospital survival rate was 93.5%. Following VA-ECMO weaning, survivors showed full recovery of the left ventricular ejection fraction (LVEF). **Conclusions**: The cardiac dysfunction observed in PMC-associated cardiogenic shock may be severe and life-threatening but appears reversible. tMCS should therefore be considered in eligible cases, as a bridge to recovery, treatment, or surgery. The reported survival rates are impressively high, suggesting possibly a substantial risk of publication bias.

## 1. Introduction

Pheochromocytoma multisystem crises (PMC) is a life-threatening endocrine emergency and represents the most severe form of presentation of pheochromocytoma and paraganglioma [1,2]. Due to its highly variable clinical presentation, delays in diagnosis are frequent, which impacts the initiation of specific treatments [2,3]. Pheochromocytoma crises may occur spontaneously or may be precipitated by trauma, anesthesia, or specific drugs [4,5]. In rare cases, PMC can evolve into a cardiogenic shock, requiring the implantation of a temporary mechanical circulatory support (tMCS) [6,7]. Veno-arterial extracorporeal membrane oxygenation (VA-ECMO) is the most frequently used temporary mechanical support and may be a lifesaving therapy in refractory cardiogenic shock [8]. In PMC-associated cardiogenic shock, VA-ECMO could be used to either initiate specific treatments and bridge the patient to recovery and weaning or to allow emergent surgery. The aim of the present review is to describe the incidence of pheochromocytoma and paraganglioma crisis associated with refractory cardiogenic shock, its pathophysiological impact on myocardial function and circulation, and the role of temporary mechanical circulatory support in its management.

### 1.1. Epidemiology

Pheochromocytoma and paragangliomas are catecholamine-secreting neuroendocrine tumors. Pheochromocytomas originate from chromaffin cells of the adrenal medulla, while paragangliomas (PGLs) arise from the extra-adrenal autonomic paraganglia [9]. Sympathetic paragangliomas are typically found in the thorax, abdomen, and pelvis, while parasympathetic PGLs are mainly located in the head and neck [5]. The combined estimated annual incidence of pheochromocytomas and paragangliomas (PPGLs) is approximately 0.46 to 0.8 per 100,000 person-years [4,5]. This condition equally affects men and women and occurs most frequently in young and middle-aged individuals [10,11]. Around 30% to 40% of PPGL cases are associated with a genetic mutation [12]. Since the fourth edition of the WHO classification of endocrine tumors, PPGLs have been classified systematically as malignant tumors, with an associated metastatic rate of 10 to 20% [13]. The clinical presentation is highly variable and ranges from asymptomatic cases to life-threatening crises. While hypertension is the most frequent cardiovascular symptom, individuals with pheochromocytoma may also experience myocardial ischemia, cardiomyopathy, aortic dissection, peripheral vascular insufficiency, arrhythmias such as sinusal tachycardia, atrial fibrillation, or ventricular premature contractions. Notably, two percent of patients with pheochromocytoma initially present with cardiogenic shock [7].

### 1.2. Pathophysiology of Cardiac Dysfunction in PPGL

The pathophysiology of cardiac dysfunction in PPGL is not yet fully understood, with the most widely accepted hypothesis being exaggerated sympathetic stimulation associated with toxic levels of circulating catecholamines that induce a form of stress cardiomyopathy [14,15,16]. Stress cardiomyopathy is a syndrome characterized by acute and reversible (<3 weeks) systolic and diastolic dysfunction [17]. The syndrome is often preceded by emotional or physical stress. The diagnosis is based on the presence of abnormal regional wall motion in the absence of any acute epicardial coronary obstruction. The regional wall motion abnormality typically extends belong the territory of a single coronary artery. Several patterns of left ventricle abnormal regional wall motion have been described, the most frequent being apical akinesia/hypokinesia (apical ballooning) associated with compensatory basal hyperkinesia, while mid-ventricular or reverse patterns occur less frequently [18]. Finally, the nature of the ventricular dysfunction must be transient [19].

Pheochromocytomas and paragangliomas exert their cardiac symptoms due to the excessive production of catecholamines, particularly norepinephrine and epinephrine, and their releases can be continuous or sporadic [16].

Catecholamines may induce microvascular spasms and/or direct myocardial toxicity, resulting in myocardial stunning and biventricular dysfunction [14,16,19]. This phenomenon, known as “Catecholamine-induced cardiotoxicity”, is a well-described entity.

Reported for the first time in 1905, its pathophysiology remained only partially understood. Back in the early twentieth century, Ziegler et al. described the occurrence of myocarditis after the injection of high doses of epinephrine in rabbits. Pearce et al. reported similar histological findings following intravenous injections of epinephrine, using an identical method [20].

While an acute increase in catecholamine level (exogenous and/or endogenous) may be lifesaving in the context of critical illness and shock, prolonged and sustained stimulation of cardiac β_1_-adrenergic receptors has negative effects on myocardial function [21]. Several hypotheses regarding myocardial dysfunction have been proposed. As stated before, the physiopathology is complex. It includes progressive myocardial desensitization to the inotropic β_1_-adrenergic stimulation (downregulation), cardiac metabolic changes, such as the stimulation of lipolysis, resulting in the deposition of neutral lipid droplets in cardiomyocytes [22,23,24,25]. In addition, prolonged exposure to catecholamines causes intracellular calcium overload and reactive oxidative stress, leading to inflammation and mitochondrial dysfunction in cardiomyocytes [26,27]. The overall result is a decrease in cardiac myofiber function with a reduced number of contractile units in a setting where systemic afterload is increased.

Catecholamine-induced cardiotoxicity has been assessed invasively and non-invasively. Endomyocardial biopsy performed on patients with pheochromocytoma-associated cardiogenic shock reported frequently ultrastructural myocardial alteration, consistent with contraction band necrosis, neutrophil infiltration, and fibrosis [28,29]. Similar findings were reported in biopsies of patients presenting Takotsubo syndrome [19].

Cardiac MRI enables us to non-invasively assess the presence of myocardial inflammation and focal fibrosis. In fact, T2-weighted imaging is used to assess if cardiac edema is present, while T1-weighted images with the use of gadolinium injection can detect hyperemia (early phase) and suggest fibrosis in late enhancement phases [30]. There is a paucity of data regarding patterns reported in pheochromocytoma-associated cardiogenic shock, including the presence of diffuse myocardial edema on T2-weighted images and intramyocardial late gadolinium enhancement [31,32].

Importantly, the toxicity of catecholamines on myocyte function appears reversible. Studies invariably reported complete recovery of the LVEF in catecholamines-induced cardiomyopathy. Similar to other forms of stress-induced cardiomyopathy, such as Takotsubo syndrome (TTS), rapid recovery of cardiac function has been reported to occur within 21 days in pheochromocytoma-associated cardiogenic shock [7,17,19].

### 1.3. Diagnosis and Evaluation

The classical triad of symptoms described in patients with PPGL includes episodic headache, tachycardia, and sweating [9,14]. However, the triad is rarely found. The paroxysmal character of the symptoms is, however, pathognomonic of this condition, and is related physiologically to the abrupt and episodic catecholamine surge. Severe cardiovascular manifestations, such as life-threatening arrhythmia, hypertensive crisis, or cardiomyopathy, account for more than two-thirds of the mortality associated with PPGL [15,33,34,35,36]. Hypertension is the most frequent sign, as fewer than 10% of the patients will be normotensive at presentation [14]. A total of 20% of patients will present with arrhythmias, supra-ventricular tachyarrhythmias being the most prevalent subtype. Life-threatening ventricular arrhythmias tend to occur in a small proportion of patients [37]. Bradyarrhythmia has been reported as well in PPGL and could be associated either with a reflex baroreceptor activation due to a hypertensive crisis or with the consequences of beta-blocker therapy.

The diagnosis could also be made incidentally in asymptomatic patients, especially with the actual widespread use of abdominal CT scans. Among adrenal incidentalomas, the prevalence of pheochromocytoma is estimated to be 1 to 5% [31].

In suspected cases, the European Society of Endocrinology recommends the measurements of plasma-free normetanephrines and metanephrines or 24 h urinary fractionated metanephrines that are consistently generated within tumor cells, regardless of the tumor’s catecholamine secretion. Adding measurements of free methoxytyramine to the plasma panel is beneficial for identifying tumors that produce dopamine [38]. These metabolites have a higher specificity than norepinephrine, epinephrine, dopamine, and vanillylmandelic acid measurements. They showed excellent sensitivity, with 97.9% for plasma-free normetanephrines and metanephrines and 93.4% for urinary-free metabolites [39]. Negative measurements allow us to effectively rule out pheochromocytoma. However, it is important to note that a positive test result only moderately increases suspicion of disease [40]. In fact, false positive tests may be associated with numerous treatments that interfere with catecholamine levels, either by increasing their secretion or inhibiting their uptake.

Elevated biomarker levels, defined as more than a twofold increase above the upper limit of the normal range, warrant further investigations. CT scans are considered the first-line imaging modality, while MRI could be recommended in specific cases. If a mass is detected, further imaging with PET-CT, 18F-DOPA PET/CT, Iodine-123-metaiodobenzylguanidine (MIBG), or ^68^Ga-DOTA–conjugated somatostatin receptor–targeting peptide (^68^Ga-DOTA-SST) may be required, depending on the characteristic of the mass, its location, biochemical phenotype, primary tumor location, as well as the availability of local imaging [38,41].

### 1.4. Management

Surgical resection remains the only definitive treatment for PPGL. However, surgery is considered a high-risk procedure and is associated with potentially severe complications. Indeed, life-threatening cardiovascular complications including hypertensive crises, arrhythmias, myocardial infarction, and cardiogenic shock may be induced by the massive release of catecholamines into the circulation during mass manipulation. Hemodynamic instability can also occur after complete mass resection and is associated with the rapid drop in plasmatic catecholamine levels.

Presurgical pharmacological treatments should be initiated one to two weeks before surgery to prevent these complications [41,42]. Nonetheless, the evidence is lacking and the recommendations are mainly based on expert opinion. The administration of a-adrenergic receptor blockers represents the cornerstone of the pharmacological management [41,42]. The most frequently medications used are phenoxybenzamine (nonselective and noncompetitive alpha-blocker) and doxazosin (selective competitive alpha1-blocker). Doxazosin is associated with fewer adverse events and a shorter half-life compared to phenoxybenzamine. In the PRESCRIPT trial, a randomized controlled study, phenoxybenzamine was more efficient than doxazosin in preventing intra-operative hypertension and hemodynamic instability. However, at 30 days, clinical outcomes were similar between the two groups [43].

In addition, tyrosine hydroxylase inhibitors could be used as an alternative or in addition to a-adrenergic receptor blockers. By inhibiting synthesis, these treatments significantly decrease plasma catecholamine levels and are considered in patients at high risk of cardiovascular complications or when monotherapy is inefficient [38,42].

Finally, calcium channel antagonists could be considered as well. Compared to tyrosine hydroxylase inhibitors, they are less expensive and widely available. Current guidelines recommend their use in patients presenting poorly controlled hypertension or when side effects preclude achieving an optimal dose regimen with alpha-blockers [38,42].

A recent pilot RCT compared the efficacy of alpha-blockers (prazosin) and calcium channel antagonist (amlodipine). Twenty patients were randomized in this monocentric study and amlodipine was reported to be more efficient than prazosin in preventing intraoperative instability [44]. However, these data must be interpreted cautiously as the study had a small sample size, was not designed as a superiority trial, and does not allow any firm conclusions to be drawn.

Beta-adrenergic-blockers must never be initiated before effective alpha-blockers, due to the risk of a hypertensive crisis in the context of unopposed stimulation of adrenergic receptors [42]. Beta-blockers are only recommended in tachycardic patients. Interestingly, Nazari et al. proposed tailoring tachyarrhythmia management in PPGL based on their biochemical phenotype (noradrenergic or adrenergic) [16]. The authors defined phenotypes by the type of catecholamine produced (epinephrine-producing PPGL vs. norepinephrine-producing PPGL) and the cardiovascular response of the patients and proposed a specific management for each phenotype.

The surgical procedure can be carried out through conventional open surgery (via anterior transabdominal, posterior, or flank routes) or through advanced minimally invasive approaches like laparoscopy or robotic-assisted surgery. Current recommendations advise that patients with PPGL presenting malignancy risk factors, such as a mass larger than 5 cm, an extra adrenal location, or some specific mutations, and those undergoing resection of a primary tumor with concurrent metastases, may benefit more from open laparotomy accompanied by lymph node dissection. The main priorities are to prevent sudden surges of catecholamines caused by pressure on the mass during removal, protect the integrity of the adrenal capsule, and limit the potential spread of tumor cells due to excessive handling [45]. Although there is no evidence for optimal preoperative hemodynamic parameters, guidelines recommend preoperative blood pressures and heart rate targets, respectively, of <130/80 mmHg and 60–70 bpm [41,42]. Finally, high saline intake and hypervolemia are commonly advised to reduce preoperative orthostatic hypotension.

The landscape of pheochromocytoma management is undergoing significant transformation, with an increasing focus on personalized medicine. For metastatic cases, innovative treatment strategies are being explored, emphasizing tailored approaches based on individual patient profiles. Emerging therapies include PARP inhibitors in combination with temozolomide, bone-seeking alpha emitters such as ^223^RaCl2, and advanced radiotherapy techniques targeting somatostatin receptors. Additionally, novel immunotherapy and radioimmunotherapy options are being investigated to enhance therapeutic efficacy [46].

Future research must shift toward prospective clinical trials to better understand the factors influencing long-term outcomes and survival in patients with PPGLs, as most current evaluations remain retrospective. A more refined risk stratification approach, integrating multi-omic analysis, is essential to personalize treatments for patients with germline mutation, in particular those carrying SDHB mutations, which are linked to higher metastatic risk. Given the high recurrence and metastasis rates in cases where surgery is not viable, the development of targeted therapies for metastatic PPGLs is critical. The expanding arsenal of chemotherapeutic agents in clinical trials offers promising avenues for improving treatment outcomes [47,48].

In parallel, optimizing preoperative risk assessment and developing precise predictive models for hemodynamic instability are fundamental to enhancing patient care. Effective strategies must be implemented to minimize complications before and during surgery. While traditional predictive models rely on clinical indicators such as tumor size, biochemical markers, and comorbidities, emerging techniques now incorporate advanced data-driven methodologies, radiomics, and genetic profiling. These innovations hold the potential to significantly improve diagnostic accuracy and treatment planning, ultimately leading to better patient management and outcomes [49].

### 1.5. Pheochromocytoma Multisystemic Crisis (PMC) and tMCS

In severe cases of PPGL, patients can present a pheochromocytoma crisis or multisystemic crisis, a rare but life-threatening endocrine emergency with a reported mortality of 13.8% [50].

Several risk factors contribute to the occurrence of PMC, such as tumor-related factors, including large tumor size, which leads to higher catecholamine secretion, SDHB mutations associated with increased metastatic potential, and bilateral tumors commonly seen in genetic syndromes like MEN2 and VHL. Various external triggers can precipitate a crisis, such as surgical manipulation or biopsy of the tumor; the use of anesthesia and certain medications (notably beta-blockers without prior alpha-blockade or corticosteroids); severe stressors like infections, trauma, pain, or childbirth; abrupt withdrawal of alpha-blockers causing rebound hypertension; and improper treatment, particularly the isolated use of beta-blockers leading to unopposed vasoconstriction. Physiological factors also play a significant role, including excessive sympathetic activation resulting in a norepinephrine and epinephrine surge, pre-existing cardiac conditions such as heart failure or stress-induced cardiomyopathy, and metabolic disturbances like electrolyte imbalance and hyperglycemia [2,51].

There is a lack of uniform diagnostic criteria, but this condition requires rapid recognition and urgent treatment. Classic clinical manifestations are nonspecific and include hypertensive crisis, cardiogenic shock, or multiple organ failure [2]. Since 2008, the use of VA-ECMO in the context of PMC-associated cardiogenic shock has been reported with increasing frequency [6,7,52,53,54]. VA-ECMO supports end-organ perfusion in patients who are refractory to optimal medical treatment, providing clinicians time to conduct further workup and plan subsequent treatment [8]. When surgery cannot be delayed or when weaning from ECLS is not possible, patients may undergo surgery under VA-ECMO support, apparently without increasing the mortality rates compared to unsupported patient [52].

### 1.6. What Is the Evidence?

For the purpose of this narrative review, we conducted a literature search of PubMed as of 16 November 2024. Medical Subject Headings (MeSH) terms used included extracorporeal circulation”, “Impella”, “pheochromocytoma”, “paraganglioma”, and “cardiogenic shock”, which were combined with Boolean “OR” and “AND”. Data from case series, retrospective studies, and systematic reviews were considered. Exclusion criteria included pediatric patients (<18 years old) and case report study designs. In addition, a language restriction was applied, with only articles in English included. Two independent investigators (A.G. and C.L.) individually performed data extraction, which was verified by a third investigator (B.A.). The following information was extracted for each individual study: study characteristics (including authors, publication year, journal, study design, recruitment period, follow-up duration, and number of patients), patient characteristics (including age, gender, comorbidities, and baseline clinical presentation), pre-ECMO left ventricular function, post-weaning left ventricular function, surgery performed under ECMO, use of LV venting strategy, in-hospital survival, and complications related to ECMO.

## 2. Results

**Figure 1:**
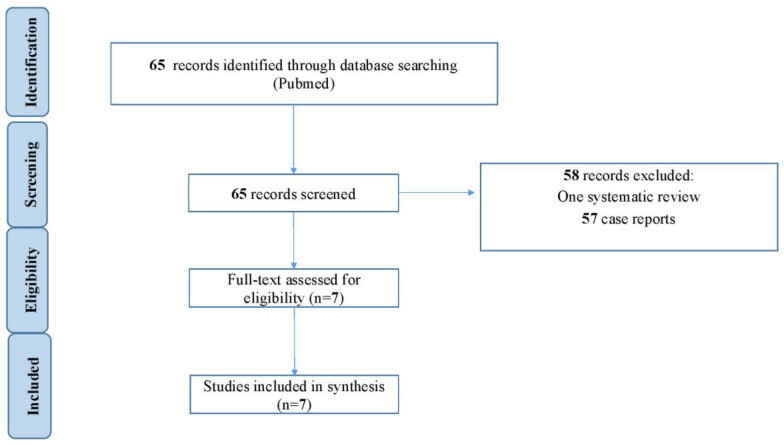
Flow diagram.

A total of 65 publications were identified in the literature search. These included one systematic review, three retrospective studies, four case series, and 57 case reports (Figure 1). Among these, seven studies—reporting on 45 patients who developed cardiogenic shock associated with PMC and required tMCS—were deemed eligible for inclusion [6,7,53,54,55,56,57].

The data were extracted and analyzed (Table 1). Study duration was reported in four studies and ranged from seven to fifteen years [6,7,53,54]. The included studies were mostly monocentric. One was performed in a high-volume and highly experienced tertiary ECLS center [6], while two retrospective studies were multi-centric and collected data from eight and fifteen hospitals [7,54]. The case series [53,55,58] had a median number of 2 (3–4) patients and retrospective studies of 11 patients (9–14) [6,7,54,57]. Patients were young, with a median age of 43 years (range 25–65) at presentation. Most cases presented with severe hemodynamic instability, blood pressure variability, and rapid progression to severe left ventricular dysfunction. Diagnosis was primarily based on CT scans and measurement of urinary or plasma metanephrines levels. Pre-ECLS left ventricular ejection fraction was reported in five studies with a median of 20% (range 5–34) [6,7,54,57,58]. Authors rarely reported right ventricular function parameters (TAPSE, S’RV, or RV FAC). Diffuse hypokinesia of the left ventricle was the most frequently reported wall motion abnormality, followed by the typical (apical ballooning) and atypical Takotsubo patterns (Table 2).

VA-ECMO was the most commonly tMCS used to stabilize patients and initiate specific pheochromocytoma treatments. A total of 20% of the included population presented with cardiac arrest and extracorporeal cardiopulmonary resuscitation (ECPR) was successfully performed in nine patients. Surgery under ECLS was performed only on 2 patients out of 45. The use of micro-axial pump devices (Impella) was rare and reported only in one patient [7]. During VA-ECMO support, LV venting was mostly provided with an intra-aortic balloon pump (IABP). IABP was reported to be used alone as a tMCS in only one study [7]. The median duration of VA-ECMO support was 4 days (range 1–7). The reported mean in-hospital survival rate was 93.5%. Following VA-ECMO or Impella weaning, a complete recovery of the left ventricular ejection fraction was observed in all survivors. Serious adverse events during ECLS were frequent, occurring in nine patients. These included lower limb ischemia requiring amputation (one case), lower limb arterial ischemia without amputation (three cases), CNS stroke (three cases), severe hemorrhage (two cases), ECLS-associated bacteremia (two cases), and KDIGO stage 3 acute kidney injury requiring renal replacement therapy (two cases) (Table 3).

## 3. Discussion

PMC is a rare condition, requiring early recognition and specific medical management.

Most patients presenting with pheochromocytoma-associated cardiogenic shock are young, with initial hypertensive crisis evolving rapidly to a hypotensive cardiogenic shock due to mono- or bi-ventricular dysfunction. As with other causes of cardiogenic shock with biventricular failure, VA-ECMO enables stabilization and treatment of refractory cases, with a survival rate reported to exceed 90%, which was associated with full cardiac recovery in survivors.

Guidelines regarding the role of ECLS in the management of pheochromocytoma-associated cardiogenic are lacking and there is a paucity of data regarding this rare condition. Indeed, we found a limited number of publications on this topic, mostly case reports and case series. Few retrospective studies were published, mostly from tertiary and ECLS-expert centers, and included only a small number of patients over a long period of study inclusion (range 7–15 years), reflecting a combination of several factors, including the low incidence of PMC associated with the possibility of underreporting and/or underdiagnosis by physicians.

In the context of cardiogenic shock and after the exclusion of frequent etiologies, physicians should have a high index of suspicion for PPGL, especially when young patients present with an atypical pattern of rapid fluctuations of blood pressure, and it may be reasonable to suggest systematic screening for pheochromocytoma in such cases.

In the included studies, VA-ECMO appeared pivotal in the management of PMC-associated refractory cardiogenic shock and 20% of the included population were ECPR patients.

Micro-axial pump devices could be considered as a tMCS, notably in cases where cardiogenic shock is associated with a predominant left ventricular failure. However, it may be required to escalate to VA-ECMO if organ failures persist, due to either inadequate support or if the patients progress to biventricular failure. tMCS appeared a reasonable strategy in well-selected cases, as complete recovery of cardiac function is expected to occur within less than 21 days [17,19]. In fact, the mean duration of support reported was four days, which is in line with data from other forms of stress cardiomyopathy requiring t-MCS support [59,60]. Surgery, which is the definitive treatment, can be performed under ECMO when weaning is not achievable and emergent surgery is required [52]. In addition, the use of tMCS usually enables rapid and significant decatecholaminization, which may play a crucial role in managing stress cardiomyopathy [15,16,61]. It is important to state that therapeutic anticoagulation can be safely held for the peri-operative period, without increasing significantly the risk of mechanical complications, such as membrane dysfunction or canula thrombosis. This was reported consistently in patients with severe bleeding during ECLS and recently in trauma patients requiring ECMO support [62]. However, it must be noted that during mechanical circulatory support, any hypertensive crisis may pose a serious risk of hemorrhagic stroke. This risk is exacerbated by therapeutic anticoagulation and the absence of baroreflex-mediated feedback, which prevents a compensatory decrease in heart rate in response to a severe increase in afterload.

The survival rates reported in the included studies and literature are remarkably high, significantly exceeding those observed in AMICS—the most common cause of cardiogenic shock—raising concerns about potential publication bias. Notably, two recent landmark RCTs in AMICS patients supported with T-MCS reported survival rates of 44.2% and 52.2% [63,64]. Beyond methodological factors, differences in underlying etiologies and prognostic variables may contribute to these discrepancies. Key prognostic risk factors in cardiogenic shock include the presence and duration of cardiac arrest before t-MCS implantation, the timing of implantation, and the potential for ventricular function recovery. This probability is influenced by the etiology of cardiogenic shock, the availability of targeted treatments such as reperfusion strategies (PCI or CABG) in AMICS, and the timing of their implementation, all of which are intrinsically linked to survival outcomes.

The findings of this narrative review are based on a low level of evidence. In fact, the included studies consist solely of case series and retrospective studies, both subject to inherent and significant methodological biases. Additionally, the small sample sizes and predominantly single-center studies further limit the generalizability of the results.

Finally, while ECMO may be a life-saving therapy, it remains associated with significant complications, including hemorrhage, infection, stroke, coagulopathy, and lower limb ischemia. As with any other indication of ECLS, strict selection and the timing of implantation are important factors to consider. The management of these patients in experienced ECLS centers is mandatory, especially in rare and complex conditions such as PPGL.

## 4. Conclusions

The cardiac dysfunction observed in PMC-associated cardiogenic shock may be severe and life-threatening, yet it appears to be reversible. The management of this specific phenotype should be individualized. tMCS appeared a reasonable strategy in well-selected cases, as the complete recovery of cardiac function is expected to occur within less than 21 days. Therefore, tMCS should be considered in eligible cases as a bridge to recovery, medical management, or surgery. The reported survival rates are impressively high, possibly suggesting a substantial risk of publication bias.

## Figures and Tables

**Table 1:** Characteristics and outcomes of included studies.

Study	Year	Design	Study Period	Number of Centers	Number of Patients	Age (IQR)	Women (%)	Hospital Survival (%)
Huang et al. [55]	2008	Case series	Not reported	1	3	36 (25–42)	33%	100%
Wu et al. [58]	2008	Case series	Not reported	1	2	41 (40–42)	0%	79%
Chao et al. [53]	2015	Case series	2005–2012	1	4	45 (25–65)	33%	100%
Hekimian et al. [6]	2016	Retrospective study	2007–2015	1	9	42 (31–51)	78%	66%
Sauneuf et al. [54]	2017	Retrospective study	2000–2015	15	14	43 (29–50)	50%	79%
Fennel et al. [57]	2023	Case series	Not reported	1	2	44 (30–58)	100%	100%
De Angelis et al [7]	2023	Retrospective study	2008–2021	8	11	45 (32–65)	66%	100%

**Table 2:** Key elements of diagnosis and clinical findings at presentation.

Study	Diagnosis	Laboratory Finding	Stress Cardiomyopathy Echographic Patterns (n Patients)	EKG Findings at Presentation	Arrhythmia at Presentation	Myocardial Biopsy
Hunag et al. [55]	Labs + Imaging	Elevated urinary metanephrines	Not reported	Not reported	SVT	Nonapplicable
Wu et al. [58]	Labs + Imaging	Elevated urinary metanephrines	Diffuse hypokinesia	ST elevation	SVT	Nonapplicable
Chao et al. [53]	Labs + Imaging	Elevated urinary metanephrines	Not reported	Not reported	VT	Nonapplicable
Hekimian et al. [6]	Imaging only (3/9)	Elevated urinary metanephrines	Typical Takotsubo pattern (2), diffuse	SVT (4), ST elevation (2),	SVT	1 patient
	Labs only (1/9)		hypokinesia (7)	ST depression (2), T waves		
	Labs + Imaging (4/9)			(1)		
	Autopsy (1/9)					
Sauneuf et al. [54]	Labs + Imaging	Elevated urinary metanephrines	Diffuse hypokinesia (8), Typical Takotsubo	Not reported	Not reported	Nonapplicable
			pattern (4), Atypical Takotsubo pattern (2)			
Fennel et al. [57]	Labs + Imaging	Elevated plasmatic metanephrines	Typical Takotsubo pattern (1), Atypical	Not reported	SVT	Nonapplicable
			Takotsubo pattern (2)			
De Angelis et al [7]	Labs + Imaging	Elevated urinary and plasmatic	Diffuse hypokinesia (2), typical Takotsubo	Not reported	SVT	Nonapplicable
		metanephrines	pattern (2), atypical Takotsubo pattern (2)			

Typical Takotsubo pattern: apical ballooning. Atypical Takotsubo pattern: reverse presentation with basal hypokinesia. SVT, supraventricular tachycardia; VF, ventricular fibrillation.

**Table 3:** Key elements associated with the use of t-MCS in pheochromocytoma and paraganglioma-associated cardiogenic shock.

Study	Type of t-MCS:	% ECPR	Median ECLS Duration (IQR)	Median Pre-ECLS LVEF (IQR)	Median Post-ECLS LVEF (IQR)	Adrenalectomy During ECLS	Complications
Huang et al. [55]	VA-ECMO	67% (2/3)	4 (2–7)	Not reported	>55%	0	None reported
Wu et al. [58]	VA-ECMO	0%	6 (4–8)	32% (30–34)	Not reported	0	1 lower leg ischemia with amputation
Chao et al. [53]	VA-ECMO	75% (3/4)	4 (1.8–6.7)	Not reported	>55%	0	2 severe lower leg compartment syndromes,
							1 early weaning with reimplantation,
							1 switch from peripheral to central cannulation
Hekimian et al. [6]	VA-ECMO	22% (2/9)	4 (1/7)	20% (5–30)	Not reported	0	1 early weaning with reimplantation and MOF
							(death), 1 septic shock (death), 2 ischemic stroke (1
							death)
Sauneuf et al. [54]	VA-ECMO	14% (2/14)	4 (3–7)	13% (10–20)	Not reported	2/14 (14%)	2 hemorrhages (1 death), 1 stroke,
							1 arterial ischemia, 1 ECLS-associated bacteriemia
Fennel et al. [57]	VA-ECMO	0%	5.5 (5–6)	<20%	>55%	0	1 ECLS-associated bacteriemia, 2 KDIGO 3 AKI
				Not reported			
De Angelis et al. [7]	VA-ECMO (5/11)	0%	Not reported	18% (10–25)	>55%	Not reported	None reported
	Impella CP (1/11)						
	IABP only (5/11)						

t-MCS: temporary mechanical support; IABP, intra-aortic balloon pump; ECLS, extracorporeal life support; LVEF, left ventricular ejection fraction, MOF, multiple organ failure; AKI, acute kidney injury.

## Abbreviation

CS	Cardiogenic shock
CT	Computed tomography
ECLS	Extracorporeal circulatory life support
FDOPA	Fluorodopa
Ga-DOTA SST	Conjugated somatostatin receptor–targeting peptide
LV	Left ventricle
LVEF	Left ventricular ejection fraction
MIBG	Iodine-123-metaiodobenzylguanidine
MRI	Magnetic resonance imaging
PET-CT	Positron emission tomography
PGL	Paraganglioma
PMC	Pheochromocytoma multisystem crisis
PPGL	Pheochromocytoma and paraganglioma
RV	Right ventricle
tMCS	Temporary mechanical circulatory support
VA-ECMO	Veno-arterial extracorporeal membrane oxygenation
